# ﻿Resolution of the *Aleiodesseriatus* (Herrich-Schäffer, 1838)-aggregate in the western Palaearctic (Hymenoptera, Braconidae, Rogadinae), with description of a new species

**DOI:** 10.3897/zookeys.1208.127135

**Published:** 2024-07-30

**Authors:** Cornelis van Achterberg, Mark R. Shaw, Jose Fernandez-Triana, Donald L. J. Quicke

**Affiliations:** 1 Naturalis Biodiversity Center, Postbus 9517, 2300 RA Leiden, Netherlands Naturalis Biodiversity Centre Leiden Netherlands; 2 National Museums of Scotland, Chambers Street, Edinburgh EH1 1JF, UK National Museums of Scotland Edinburgh United Kingdom; 3 Canadian National Collection of Insects, 960 Carling Avenue, Ottawa, Ontario, K1A 0C6, Canada Canadian National Collection of Insects Ottawa Canada; 4 Integrative Insect Ecology Research Unit, Department of Biology, Faculty of Science, Chulalongkorn University, Phayathai Road, Pathumwan, BKK 10330, Thailand Chulalongkorn University Pathumwan Thailand

**Keywords:** *Aleiodespseudoseriatus* sp. nov., *Aleiodesseriatus*, *Atolmisrubricollis*, *Eilemagriseola*, molecular barcodes, morphology, taxonomy

## Abstract

Two European species are recognised and characterised within the traditional *Aleiodesseriatus* species concept, based initially on DNA barcoding but with supporting, although slight and sometimes unreliable, morphological differences. *Aleiodespseudoseriatus***sp. nov.** is described and a neotype is designated for *Rogasseriatus* Herrich-Schäffer, 1838. Specimens from the Russian Far East were also DNA barcoded and were found to belong to a new species distinct from the two European taxa. The two European species were found to use different lithosiine hosts.

## ﻿Introduction

At the time of the first part of our revision of the western Palaearctic species of *Aleiodes* Wesmael, 1838 (van Achterberg and Shaw 2016) we had cytochrome *c* oxidase subunit 1 sequences (CO1; DNA barcodes) for enough European specimens to be confident that there were two different species hidden under the name *A.seriatus* (Herrich-Schäffer, 1838), but not enough to engage in a fruitful analysis. We therefore took the decision to treat *A.seriatus* as an aggregate, and gave minimal data and illustration of it. In the intervening years, however, we have been able to DNA barcode many more specimens from various European localities, and can now present the results of combining the molecular (CO1) dataset with morphological analysis of the barcoded specimens (all but one in the National Museums of Scotland, NMS; the exception (MRS819) is in the Natural History Museum, London (NHMUK)) and other material, especially in both NMS and the Naturalis Biodiversity Center, Leiden (RMNH), to give diagnoses and descriptions of the two cryptic species.

## ﻿Materials and methods

### ﻿Morphology

Morphological analysis of molecular barcoded specimens (35 females, 26 males and one unsexed prepupa) was undertaken over a two-day period, and the characters found were tested on a further 212 specimens (including 84 females) from a wide range of European countries immediately afterwards. Series from single localities at which only one species occurred were instrumental (for *A.seriatus*: long series from England, Cambridgeshire, Chippenham Fen in the period 1983–1985 (27♀, 9♂); Czech Republic, České Budéjovice, Černiš wetland in 2009 (13♀, 21♂); and France, Var, Callas from 2017 to 2023 (14♀, 19♂).For the proposed new species, *A.pseudoseriatus*, we examined a shorter series from Cumbria, England and several sites from Sweden in the period 2004–2017). The analysis was later extended to include material from RMNH. The countries of origin of the specimens used (which are mostly in NMS or RMNH except for the long series of *A.seriatus* from Černiš wetland in the Institute of Entomology, České Budéjovice, Czech Republic, IECB, and a smaller number of other specimens as indicated) is given in the species accounts below.

Morphological terminology follows van Achterberg (1988, 1993) and van Achterberg and Shaw (2016), including the abbreviations for wing venation. Measurements are taken as indicated by van Achterberg (1988): for the length and the width of a body part, the maximum length and width are taken, unless otherwise indicated. The length of the mesosoma is measured from the anterior border of the mesoscutum to the apex of the propodeum, and of tergite I from the posterior border of the adductor to the medio-posterior margin of the tergite.

Observations and descriptions were made under an Olympus SZX11 stereomicroscope. Photographic images were taken with a Canon 5Ds 50.6-megapixel camera combined with a Canon MP-E 65 mm f/2.8 1–5× Macro lens, Laowa Macro Twin flash KX-800 and an electronic WeMacro Z-stepper rail. The photos were stacked with Helicon Focus 7 software. Some photographs were taken with a Keyence (VHX-7000) digital microscope.

### ﻿Depositories

**BZL**Oberösterreichisches Landesmuseum, Biologiezentrum, Linz, Austria

**IECB** Institute of Entomology, České Budéjovice, Czech Republic

**MSC** M. Schwarz collection, Linz, Austria

**MTMA**Hungarian Natural History Museum, Budapest, Hungary

Natural History Museum, London, England

**NMS**National Museums of Scotland, Edinburgh, Scotland

**RMNH**Naturalis Biodiversity Center, Leiden, Netherlands

**ZSM**Zoologische Staatssammlung, Munich, Germany

### ﻿Molecular and phylogenetic methods

Specimens were DNA barcoded at the Biodiversity Institute of Ontario, University of Guelph, using their standard methods (Hrcek et al. 2011), generating an approximately 650 base pair, 5’ region cytochrome *c* oxidase subunit 1 (COI). Sequence alignment was trivial as there were no indels.

Sequence data were partitioned according to the three codon positions, and a maximum likelihood (ML) tree was constructed using RAxML-NG (Kozlov et al. 2019), with the GTR+FC+4Gm+BU model applied to each partition, and a full bootstrap also performed. The most likely tree was visualised using FigTree version 1.4.4. (Rambaut 2018). Three species which collectively bracket *A.seriatus* in the best tree presented by van Achterberg et al. (2020) were included as outgroups. A haplotype network was generated for the *A.seriatus* aggregate sequences using the program PopART (Leigh and Bryant 2015).

## ﻿Results

### ﻿Molecular results

**Figure 1.**
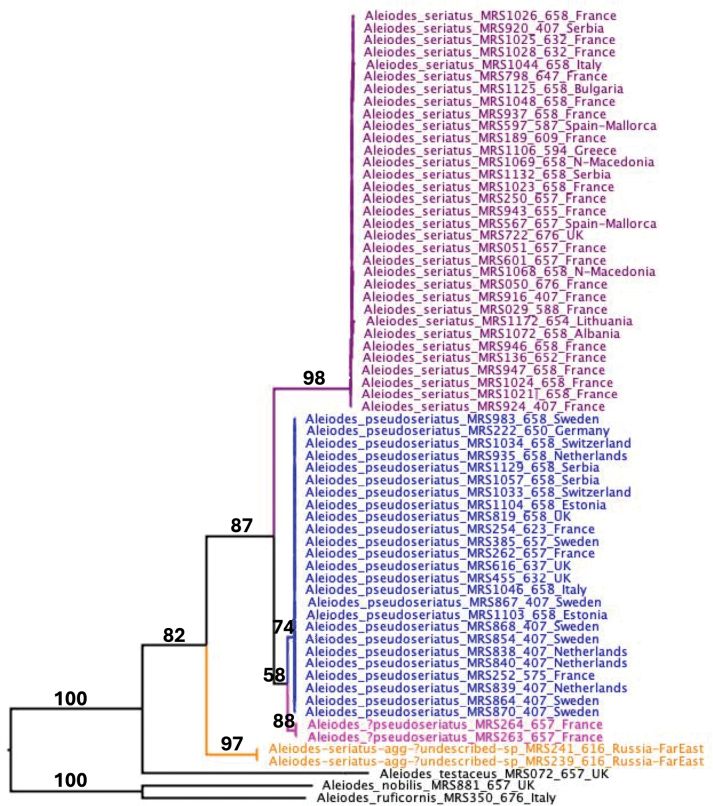
Maximum likelihood tree based on all DNA barcode sequence data for taxa included in this paper, rooted using sequences from three related *Aleiodes* species, with full bootstrap support values for selected branches.

**Figure 2.**
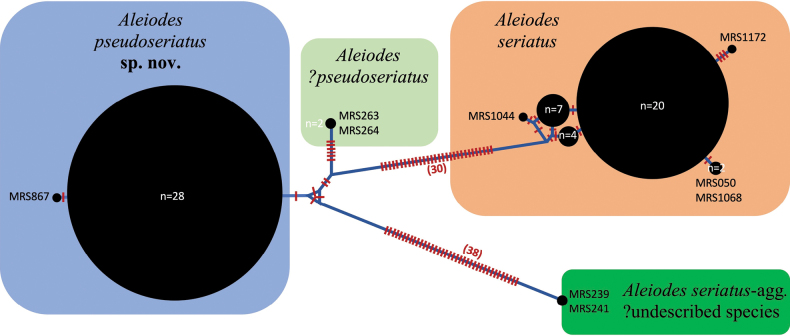
Median neighbour joining haplotype network for *A.seriatus* aggregate specimens.

The maximum likelihood phylogeny obtained (Fig. 1) shows that the sequenced individuals of the *A.seriatus*-aggregate form three clusters, with those from *A.seriatus* s. str. and the two specimens from the Russian Far East showing no, or very little, intraspecific sequence variation, and those of *A.pseudoseriatus* sp. nov. forming two small clusters but with little separation between them. *Aleiodesseriatus* s. str. was strongly supported (98% bootstrap) as being separate from the combined clusters representing *A.pseudoseriatus* sp. nov. and two putative French members of this species, though the latter two only received 58% bootstrap support. Consideration of the haplotype network (Fig. 2) shows that *A.seriatus* s. str. and the main cluster of *A.pseudoseriatus* sp. nov. sequences differ by a minimum of 34 base pairs. The two French specimens labelled as A.?pseudoseriatus differ from *A.pseudoseriatus* sp. nov. and *A.seriatus* by a minimum 10 and 30 base pairs, respectively.

### ﻿Morphology and descriptive taxonomy

The characters found were sufficient to provide unequivocal identification – at least for females – of about 90% of individual specimens, and much more than that if series are available. Males are less easy to determine with certainty, but (even if not barcoded) can often be confidently associated with females from the same locality. However, we have found one locality (France: Côte-d’Or, Abbaye de la Bussière) where light-trapping produced a good series of both species with seemingly identical body markings (some of each subsequently barcoded) on the same night.

### ﻿Diagnosis of the *Aleiodesseriatus* aggregate (cf. van Achterberg and Shaw 2016)

It should be noted that the species aggregate here defined applies to the typical species *A.seriatus* (Herrich-Schäffer) and its nearest cryptic relatives, and is not the same as the broader concept of the *A.seriatus* species-group as expressed by Marsh and Shaw (1998), Fortier and Shaw (1999), Townsend and Shaw (2009), Shimbori and Shaw (2014), and Shaw et al. (2020), which has been applied more broadly to a morphologically-defined large assemblage of New World *Aleiodes* species with a margin of flat setae along the inner apex of the hind tibia.

Antennal segments of ♀ (35–)44–55, of ♂ (40–)46–58; length of malar space of ♀ 0.3–0.4× (of ♂ 0.25×) height of eye in lateral view (Figs 14, 18); OOL 0.8× diameter of posterior ocellus; temple narrow (Figs 13, 25); surroundings of veins 1-M and 1-SR of fore wing more or less infuscate (Figs 5, 19, 22, 23, 26, 28); inner apex of hind tibia with comb (Fig. 10); metasoma of ♀ maculate (Fig. 8) but in pale specimens less developed or absent (Figs 28, 29); fourth tergite of ♀ pale (ivory) yellowish latero-posteriorly, in ♂ usually infuscate or this tergite uniformly pale brown; base of hind tibia usually narrowly dark brown (Fig. 26); length of hind femur of ♀ 5.1–6.5× its width (of ♂ up to 8×); fourth tergite gently folded laterally, without acute lateral crease or this only anteriorly developed, although rarely present as a simple, non-lamelliform crease to apex of tergite; precoxal area, epicnemial area and propodeum laterally, rugose; fourth tergite superficially transversely rugulose or aciculate; setose part of ovipositor sheath 0.6× as long as hind basitarsus. The patterning of the metasoma is characteristic but very variable in extent, and especially in pale specimens it may be absent (Figs 28, 29). Specimens with reddish and (almost) unmarked metasoma are rather frequent in southern populations of *A.seriatus*. Poorly marked forms (but almost never with completely pale metasoma) of *A.pseudoseriatus* sp. nov. seem less frequent and then have the second and third tergites more ivory than reddish.

### ﻿Key to West Palaearctic species of the *A.seriatus* aggregate

**Table:** 

1	Subbasal cell of fore wing setose apically (**aa** in Fig. 21); pterostigma distinctly pale yellowish antero-basally (Figs 22, 26, 28, 29)	***A.seriatus* (Herrich-Schäffer, 1838) [ca 80% of ♀, 50% of ♂, specimens**]
–	Subbasal cell of fore wing with glabrous patch apically (**a** in Fig. 20); pterostigma variable, often with less developed pale yellowish patch or entirely brown antero-basally (Figs 3, 5)	**2**
2	Glabrous patch in apical part of subbasal cell tending to be narrow and extending basad nearest to posterior margin (i.e., alongside 1-1A); pterostigma usually distinctly pale yellowish antero-basally (Figs 22, 26, 28); **if** (♀) hind femur partly dark brown laterally **then** also so ventrally; fourth antennal segment brown or yellowish brown ventrally, similar to scapus; vein 1-M of fore wing of ♂ and surrounding area often slightly less darkened	***A.seriatus* (Herrich-Schäffer, 1838) [ca 20% of ♀, 50 of ♂, specimens**]
–	Glabrous patch tending to be wider, when small or narrow more nearly equidistant between veins 1-CU1 and 1-1A (Fig. 20); pterostigma usually dark brown or brown antero-basally (Fig. 3), rarely yellowish in ♀ (but usually suffusedly so in ♂) (Figs 18, 19); **if** (♀) hind femur partly dark brown, **then** usually paler ventrally than laterally; fourth antennal segment dark brown (Figs 3, 15), **if** less brown **then** usually still darker than scapus ventrally (Fig. 18); vein 1-M of fore wing of ♂ and surrounding area often slightly more darkened (but variable in both species)	***A.pseudoseriatus* sp. nov.**

#### ﻿ Aleiodes pseudoseriatus

Taxon classificationAnimalia

van Achterberg & Shaw sp. nov.

612CAB63-C9BA-5D4F-A944-F1E8E5B7FE57

https://zoobank.org/C9AA7A50-C166-4CD1-94F8-E985995DE9EF

Figs 3–4 , 5–17 , 18–19 , 20

##### Type material.

***Holotype***, ♀ (NMS), “**Italy**, Veneto, Vittoria Veneto (VT), Frazione di fais, 46.017N 12.274E (WGS48), 450 m, 18.vii.2016, [at] UV light, D. Dal Pos”, “MRS *Aleiodes* DNA 1064”, “DNA COI worked”. ***Paratypes***: 1 ♂ (BZL), **Austria**: Bad Ischl, OÖ [= Oberösterreich], Höherstein, 820 m, lux SW-wand, Forststrasse, N.47.686° E13.689°, 3.vii.2010, N. Pöll”; 1 ♂ (MSC), A[ustria]: Oberösterreich, 13 km SSW Reichraming, Krahlalm, 47°46'N, 14°23'E 22.vi.2011, 680–850 m, M. Schwarz”; 1 ♂ (BZL) “A-OÖ [= Austria: Oberösterreich], Linz-Urfahr, Pragerstrasse, N48.19.16 E14.17.36 18–19.vii.2013, Trefenthaler”; 1 ♂ (BZL), id., but “KGA Riesenhof, Parz 60 E14.16.15 N48.19.06, 5–8.ix.2013”; 1 ♂ (RMNH), “**Belgium**: Liège, Mt. Rigi, 650 m, 1–2.viii.1986, at light, C. Bank, RMNH”; 2 ♂ (RMNH), id., but 2.viii.1986, C. v. Achterberg; 1 ♀ (NMS), “**Bulgaria**: W Stara Planina Mts, confluence of Penkova and Berkovska rivers, 558 m, N43.2233 E023.07596, 10.ix.2021, S. Beshkov & A. Nahirnić-Beshkova”; 1 ♂ (BZL), “CZ [= **Czech Republic**]: Bohemia, C. Budéjovice, D. Voda, N48°58’ E14°32’ 470 m, M. Halada 10.vii.2001”; 1 ♀ (NMS), “[**England**:], Cumbria, Howe, Whitbarrow, [at] MV light, 24.viii.[19]95, M.R. Shaw”; 1 ♂ (NMS), “Cum[bria], Roudsea Wood, at light, 15.vii.[20]06, M.R. Shaw”, “MRS *Aleiodes* DNA 455”, “DNA CO1 worked”; 1 ♂ (NMS), id., but “MRS *Aleiodes* DNA 616”, “DNA CO1 worked”; 1 ♂ (NHMUK), “England: E. Kent, West Wood, TR1426143868, MV light, 29.viii.2011”, “MRS *Aleiodes* DNA 819”, “DNA CO1 worked”; 1 ♀ (NHMUK), “England, Cornwall, Ding Dong, Tredinnick Stack, SW444348 MV light trap, J. Herbert, BMNH(E) 2012-41”; 1 ♂ (NHMUK), “[England]: Hen Wood, SU6522 Hants VC11, 23.vii.2013 MV”; 1 ♀ (NMS), “**Estonia**: Piargu, Raplamaa Farmland, [N]59.122167, [E]24.831745, 9.ix.2019 MV light, Kaido Kärner”, “MRS *Aleiodes* DNA 1103”, “DNA CO1 worked”; 1 ♂ (NMS), id., but “MRS *Aleiodes* DNA 1104”, “DNA CO1 worked”; 1 ♀ (NMS), “**France**: Côte d’Or, Abbaye de la Bussière, La Bussière-sur-Ouche, at light, 19.vii.2003, M.R. Shaw”, “MRS *Aleiodes* DNA 262”, “DNA COI worked”; 1 ♀ (NMS), id., but “MRS *Aleiodes* DNA 254”, “DNA CO1 worked”; 1 ♀ (NMS), id., but “MRS *Aleiodes* DNA 252”, “DNA CO1 worked”; 1 ♂ (RMNH), “France: Finistère, Forêt du Cranou, 7 km E [of] le Faou, on *Taxus*, 27.vi.1988, M.J. Gijswijt”; 1 ♀ (RMNH), “France: Doubs, RN Lac de Remoray, 16.viii.2009, Mal. tr[ap] 3, 948242/6634536, H. Gens, RMNH’23”; 1 ♀ (NMS), “**Finland**: Oulu, Ketolanoja, Muhos, Mal.tr. 5–19.viii.[20]05, N. Laurenne”; 1 ♀ (NMS), “**Germany**: Bayerswald, 2001, M. Kuhlmann”, “MRS *Aleiodes* DNA 222”, “DNA CO1 worked”; 1 ♂ (RMNH), “Germany: Thüringen, NP Hainich, nr Eisenach, [reared] from *Fagussylvatica* stems, 12.vi-3.vii.2008, M. Gossner, RMNH’08”; 1 ♀ (ZSM), “[Germany]: Ober Bayern, Garmisch, 12–1300 m, 10.viii.1936, E. Bauer”; 2 ♀ (ZSM), “[Germany]: Ebenhausen, Isart, viii.[19]40, K.V. Rosen”; 7 ♂ (MTMA), “**Hungary**, Nógrád m., Bátonyterenye (Kistererenye), Csente, Kertvárosi kert”, “48.0074992°/19.8180737° [= 20.viii–9.ix.2016], P.G. Sulyán, lámpázás (6)”; 1 ♂ (MTMA), id., but “[= 24.ix–30.ix. 2016]... lámpázás (8)”; 1 ♂ (MTMA), id., but [= 15.x.2016]...lámpázás (9)”; 1 ♀ (NMS), “[**Ireland**]: Wexford, 5.vii.[19]02, J.J.F.X. King”; 1 ♀ (NHMUK), “[Ireland]: Kilkea Deerpark, Co, W[e]x[ford], 4.vi.1937, A.W. Stelfox”; 3 ♀ + 2 ♂ (NMS), “**Italy**: Veneto, Riserva Naturale Integrale Bosco Nordio, Chioggia, 45.122N 12.260E, 28.vii.2016, D. Dal Pos”; 2 ♂ (NMS), id., but “3.vi.2016”; 1 ♂ (NMS), “**Netherlands**: Noord Holland duinreservaat, Egmond aan Zee, MV 8.vii.2016, M.R. Shaw”, “MRS *Aleiodes* DNA 838”, “DNA CO1 worked”; 1 ♂ (NMS), id., but “MRS *Aleiodes* DNA 839”, “DNA CO1 worked”; 1 ♂ (NMS), id., but “MRS *Aleiodes* DNA 840”, “DNA CO1 worked”; 2 ♂ (NMS), id., but no DNA labels1 ♂ + 1 ♀ (RMNH), “Netherlands: Gld, Tongeren, 3.ix.1991, B. v. Aartsen”; 1 ♀ (RMNH), id., but 9.vii.1989, C.J. Zwakhals; 1 ♀ (RMNH), “Netherlands: LI, Brunssum-Treebeek, c. 100 m, 50°56'17"N, 5°56'58"E, garden, at light, 25–31.vii.2018, G. Lommen, RMNH”; 1 ♂ (RMNH), id., but 3–10.vi.2018; 1 ♀ (RMNH), “[Netherlands: UT,] 3bergen [= Driebergen], Six [c. 1860]”; 1 ♀ (RMNH), “[? Netherlands, Hilvarenbeek], H.B., 3.vii”; 1 ♂ (RMNH), “Nederland: Gld, ‘t Harde, 16.viii.1993, B. v. Aartsen”; 1 ♀ (RMNH), “Netherlands[: FR], Fochtelo, 4.ix.2001, B. v. Aartsen; 1 ♀ (RMNH), “Netherlands: DR, Borger, Boswachterij Borger, UTM LD, 495693, SBB-vak 26, 25–28.vii.1993, Mal. tr[ap], L. Witmond”; 1 ♀ (RMNH), “Netherlands: NB, Tilburg, Kaaistoep, at light, 18.vii.2017, 128.8–394.6, T. Peeters, RMNH’18”; 1 ♀ (RMNH), “[Netherlands:] Gld, Epe, de Dellen, 19.vii.1994, B. v. Aartsen”; 1 ♂ (RMNH), “[Netherlands:] OV, Hasselt, Stadsgaten, 24.vii.1994, B. v. Aartsen”; 3 ♀ (RMNH), “Netherlands: NB, Achtmaal, O. Bluisse Heide, MT, R.D. 97–386, 5.viii.2015, E. Brosens”; 1 ♂ (RMNH), id., but 15.viii.2015; 1 ♀ (NMS), “**Norway**: RY Hølland, 58.52445N 5.83518E, 17.vii–2.vii.2020 Mal. tr. A.T. Mjøs”; 1 ♂ (NMS), “**Serbia**: Kasan, N of Prepollent, 1256 m, 43°19'35"N, 19°96'44"E, 3.vii.2019, C.W. Plant”, “MRS *Aleiodes* DNA 1057”, “DNA COI worked”; 1 ♀ (NMS), “Serbia: Tzaribrod (Dimitrovgrad) distr., Vištni Kamen above Bačevo Village, 763 m, N43.0271, E022.8239 11.viii.2021, S. Beshkov & A. Nahirnić-Beshkova”, “MRS *Aleiodes* DNA 1129”, “DNA CO1 worked”; 1 ♀ + 3 ♂ (NMS), “Serbia: Suva Planina, Preslap, 1186 m, N43.19473 E022.24400, 30.vi.2021, S. Beshkov & A. Nahirnić-Beshkova”; 1 ♀ (NHMUK), “Yugoslavia, **Slovenia**, Postojne, 24.vii, R.L. Coe”; 1 ♀ (RMNH), “Espana [= **Spain**:] Huesca, Torla, 1035 m, 8–26.vii.1974, J. Wolschrijn”; 1 ♀ (NMS), “**Sweden**: Bohuslän, Tossene, Åby, MV, 9.vii–13.viii.2013, N. Ryrholm”, “MRS *Aleiodes* DNA 864”, “DNA CO1 worked”; 1 ♂ (NMS), id., but “14.viii–21.xi.2013” and no DNA labels; 1 ♂ (NMS), “Sweden: Bohuslän, Tossene, Stora Hultet MV, 8.viii–21.xi.2013 N. Ryrholm, “MRS *Aleiodes* DNA 867”, “DNA CO1 worked”; 1 ♂ (NMS), id., but “MRS *Aleiodes* DNA 868”, “DNA CO1 worked”; 1 ♂ (NMS), id, but “28.v–5.viii.2013” and no DNA labels; 1 ♂ (NMS), “Sweden: Gästrikland, Staffen, Grinduga, MV, 23.vii–9.9.2013 N. Ryrholm”, “MRS *Aleiodes* DNA 870”, “DNA CO1 worked”; 1 ♂ (NMS), “Sweden: Ha[lland],Ysby Perstorp, 1–8.viii.2004, N. Ryrholm, NMSZ 2004.167”, “MRS *Aleiodes* DNA 385”, “DNA CO1 worked”; 2 ♂ (NMS), id., but no DNA labels; 1 ♂ (NMS), “Sweden: Skåne, Ö Hoby, Spraggehusen, MV, 1.ix–30.x.2013 N. Ryrholm”, “MRS *Aleiodes* DNA 854”, “DNA CO1 worked”; 2 ♂ (NMS), id., but no DNA labels: 1 ♂ (NMS), “Sweden: Skåne, Spraggehusen, MV, 20.v–16.vii.2017 N. Ryrholm/C. Källender”, “MRS *Aleiodes* DNA 983”, “DNA CO1 worked”; 2 ♀ + 4 ♂ (NMS), “Sweden: Skåne, Käseberga, Käseberga,17.vii–14.ix.2013, N. Ryrholm”; 1 ♀ (NHMUK), “Sweden: Sk[åne], Degaberga, 8.vii.1938, D.M.S. P[erkins] & J.F. P[erkins], B.M. 1938-414”; 1 ♀ + 1 ♂ (NHMUK), id., but “10.vii.1038”; 1 ♀ (NHMUK), id., but “14.vii.1938”; 3 ♀ (NHMUK), “Sweden: Skåne, Löderup, 27.vii.1938, D.M.S. P[erkins] & J.F. P[erkins], B.M. 1938-414” 1 ♀ (NMS), **Switzerland**: BE, Lenk, Brandegg, 1540 m, 29.vi–3.vii.2019, M.R. Shaw”, “MRS *Aleiodes* DNA 1033”, “DNA CO1 worked”; 1 ♀ (NMS), id., but “MRS *Aleiodes* DNA 1034”, “DNA CO1 worked”; 1 ♀ (NHMUK), “Switzerland: Grindelwald, viii.1937, G. Nixon”;1 ♀ (RMNH), “CH [= Switzerland]: Lauerz, SZ, Schuttwald, 480 m, 8.viii.1990, Lf, L. Rezbanyai-Reser”; 1 ♂ (RMNH), id., but 26.vi.1990; 1 ♂ (RMNH), id., but 11.ix.1991; 1 ♂ (RMNH), id., but Sägel (Ried), 455 m, 24.vii.1990. Most unassociated males are considered too doubtfully determined to be treated as paratypes.

##### Molecular data.

We have DNA barcoded material from England, Estonia, France, Germany, Italy, Netherlands, Serbia, Sweden and Switzerland (see Fig. 1).

##### Biology.

The record (Fahringer 1934) of *A.* “*vittiger*” from *Atolmis* (as *Gnophria*) *rubricollis* (Linnaeus) (Lepidoptera: Erebidae, Arctiinae, Lithosiini) is presumed to relate to this species, but we have not seen a reared specimen ourselves except for one partially formed adult extracted from a mummy of this host from Austria that is, unfortunately, not in good enough condition to be determined unequivocally as *A.pseudoseriatus*. However, we have barcoded the dead parasitoid prepupa (MRS935) from a failed mummy of this moth from the Netherlands and it clusters in the tree unequivocally with *A.pseudoseriatus*. Also, at a site in S. Cumbria, England where *A.pseudoseriatus* is the only one of the two relevant *Aleiodes* we have found (and barcoded), we have on several occasions obtained mummies of *A.rubricollis* that must undoubtedly have harboured *A.pseudoseriatus*, though unfortunately, none survived to produce adults of the parasitoid. The host is increasingly widely distributed and abundant in Europe, and its larva feeds on algae on (often dead) twigs of trees, perhaps with a special liking for conifers, from about July into October. It overwinters as a pupa (unlike *Eilemagriseola*), so in this case the parasitoid overwinters in the host mummy and has proved to be difficult to rear. To judge from their behaviour in captivity, parasitised *Atolmisrubricollis* larvae probably descend from trees to mummify in the litter rather than the mummy forming on twigs. *Aleiodespseudoseriatus* is univoltine with a flight time from the very end of June to September.

A female paratype (not barcoded but confidently determined and from the area in England (S. Cumbria) where only *A.pseudoseriatus* has been found (and barcoded)), was offered cultured larvae of the lithosiin arctiine *Eilemagriseola* (Hübner) at various stages of growth in viii.1995, by day and at dusk when she was more active, but apart from very brief antennation on a minority of occasions she showed no interest in them.

##### Diagnosis.

**Figures 3, 4.**
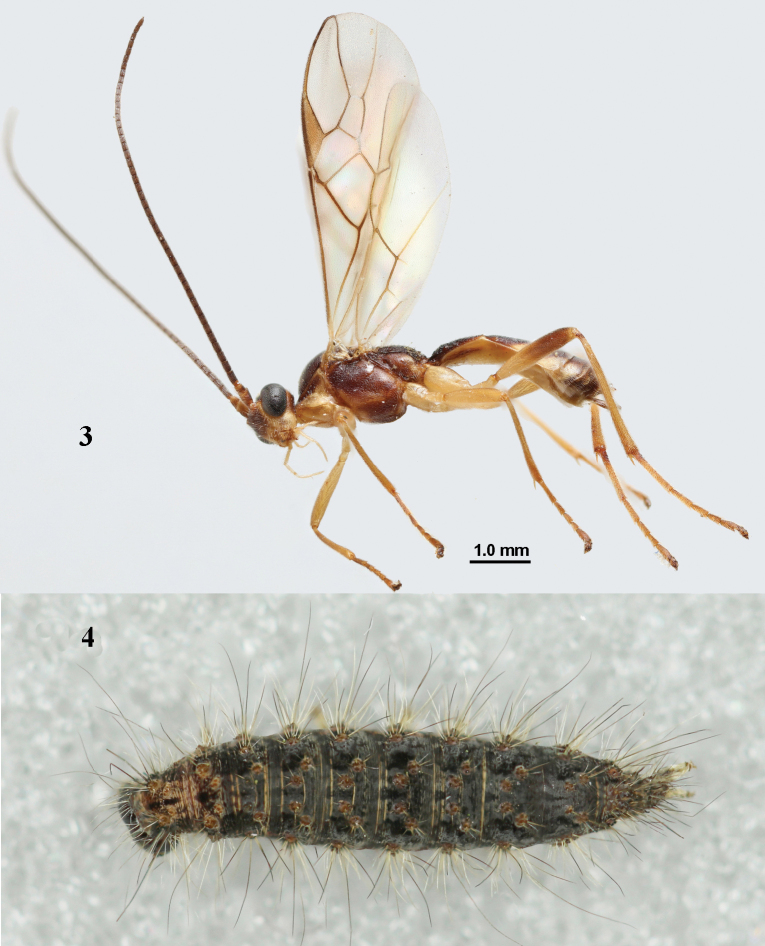
*Aleiodespseudoseriatus* sp. nov., paratype, ♀ (**3**), and mummified caterpillar of *Atolmisrubricollis* (Linnaeus) with *A.pseudoseriatus* larva within (**4**), both from England, S. Cumbria, Whitbarrow. **3** habitus, lateral view **4** mummy, dorsal view.

**Figures 5–17.**
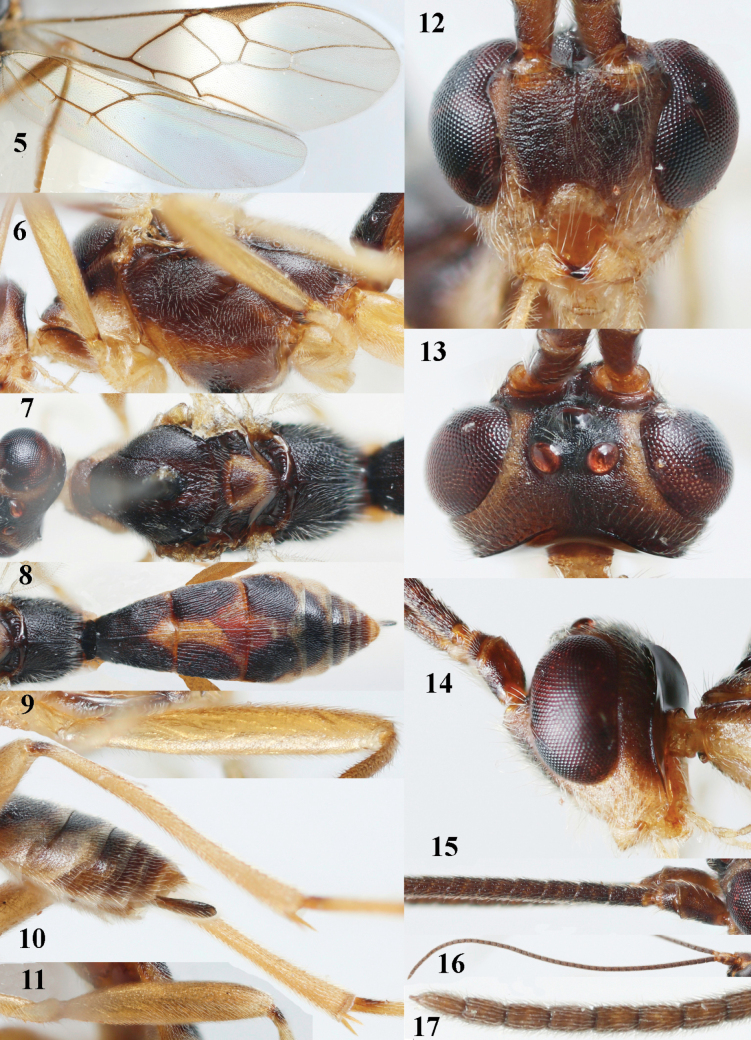
*Aleiodespseudoseriatus* sp. nov., holotype, ♀, Italy, Vittoria Veneto, but 17 of paratype ♀ from England, Whitbarrow. **5** wings **6** mesosoma, lateral view **7** mesosoma, dorsal view **8** propodeum and metasoma, dorsal view **9** fore femur, lateral view **10** ovipositor sheath **11** hind femur, lateral view **12** head anterior **13** head, dorsal view **14** head, lateral view **15** base of antenna **16** antenna **17** apex of antenna.

**Figures 18–19.**
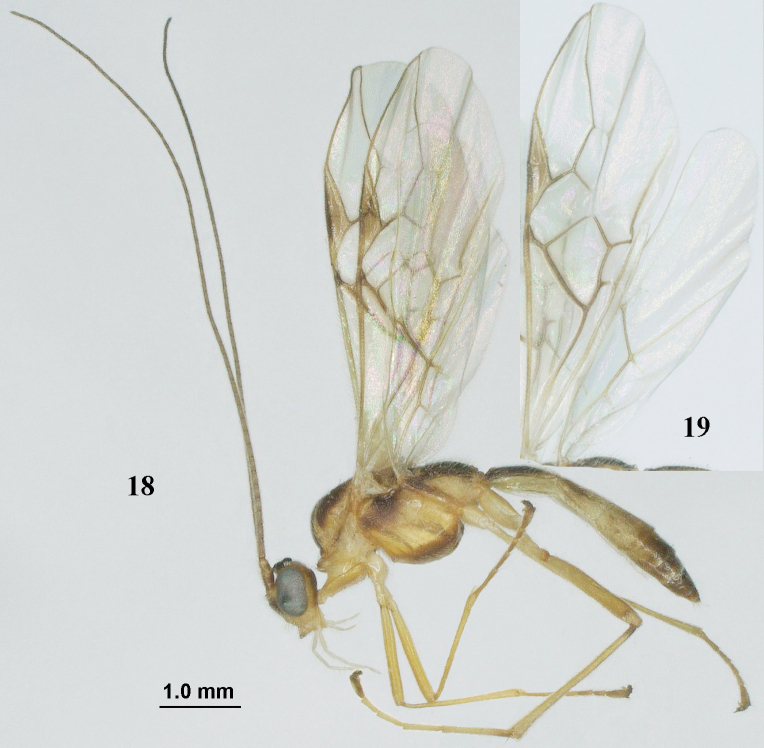
*Aleiodespseudoseriatus* sp. nov., paratype, ♂, England, S. Cumbria, Roudsea Wood. **18** habitus, lateral view **19** wings.

**Figures 20–25.**
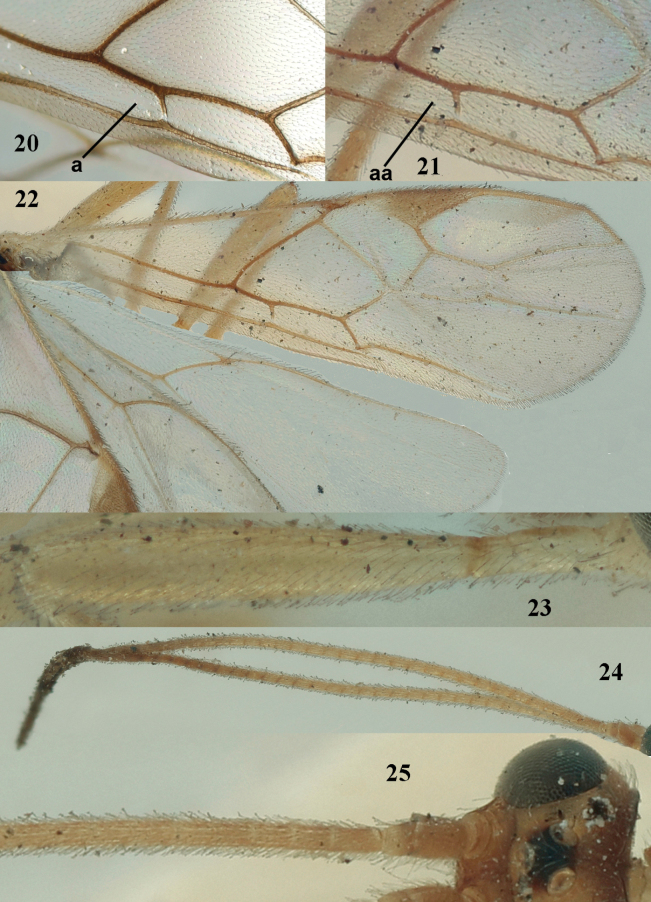
*Aleiodesseriatus* aggregate: holotype of *A.pseudoseriatus* sp. nov. (**20**), *Aleiodesseriatus* (Herrich-Schäffer), lectotype of *A.vittiger* Wesmael, ♀, Belgium (**21–25**) **20, 21** detail of distal half of subbasal cell of fore wing (“a” indicating comparatively large glabrous patch and “aa” a minute glabrous patch) **22** wings **23** fore femur **24** antennae **25** base of antenna. Photographs by Julian Lalanne except 20.

Subbasal cell of fore wing with small glabrous patch apically (**a** in Fig. 20); pterostigma variable, often with less-developed pale yellowish patch or entirely brown antero-basally (Figs 3, 5); hind femur of ♀ usually 4.7–5.5× longer than wide; pterostigma usually dark brown or brown antero-basally, rarely yellowish (Figs 5, 18, 19); **if** ♀ hind femur partly dark brown, then usually paler ventrally than laterally; fourth antennal segment dark brown (Figs 3, 15), if brown then darker than scapus ventrally (Fig. 18); vein 1-M of fore wing of ♂ and surrounding area often darker than in *A.seriatus*. On average with about 3 more antennal segments than *A.seriatus* in both sexes. We have also seen the holotype of *Rogaskuslitzkyi* Tobias, 1976, from Azerbaijan and believe it can be ruled out to belong to *A.pseudoseriatus* (see also notes on barcoded specimens from Primorsky Krai below).

##### Description.

Holotype, ♀, length of fore wing 5.2 mm, of body 5.9 mm.

***Head*.** Antenna incomplete, but according to label originally with 47 segments, length of antenna in ♀ paratype from England 1.3× fore wing and its subapical segments medium-sized (Fig. 17); frons granulate and distinctly depressed laterally; OOL 1.5× diameter of posterior ocellus, granulate and matt; depression near posterior ocellus granulate; vertex largely granulate-coriaceous, rather dull; clypeus coriaceous; ventral margin of clypeus depressed (Fig. 12); face granulate but dorsally rugulose; width of hypoclypeal depression 0.4× minimum width of face (Fig. 12); length of eye 3.6× temple in dorsal view (Fig. 13); vertex behind stemmaticum rugulose-granulate; clypeus largely above lower level of eyes; length of malar space 0.3× length of eye in lateral view.

***Mesosoma*.** Mesoscutal lobes finely granulate-coriaceous, matt; precoxal area of mesopleuron rugulose but posteriorly absent, and area above it finely granulate; metapleuron densely granulate and ventrally rugose; metanotum with short median carina anteriorly and distinct depression posteriorly; scutellum finely granulate; propodeum rather long and flat, granulate anteriorly and densely rugose posteriorly, medio-longitudinal carina complete, and without protruding carinae laterally.

***Wings*.** Fore wing: r 0.4 × 3-SR (Fig. 5); 1-CU1 horizontal, 0.7 × 2-CU1; r-m 0.3 × 3-SR; second submarginal cell medium-sized (Fig. 5); cu-a inclivous, straight; 1-M straight posteriorly; 1-SR as wide as 1-M; surroundings of M+CU1, 1-M and 1-CU1 setose, but subbasal cell with small glabrous patch apically (a in Fig. 20). Hind wing: marginal cell parallel-sided, its apical width 1.1× width at level of hamuli (Fig. 5); 2-SC+R as long as wide; short m-cu present anteriorly; vein 2-1A absent (Fig. 5); M+CU:1-M: 1r-m = 30:18:18.

***Legs*.** Tarsal claws rather robust, bristly setose and very finely yellowish pectinate; hind coxa rather shiny and only very superficially micro-sculptured, dorsally granulate; hind trochantellus rather slender (Fig. 11); length of hind femur and basitarsus 5.1 and 8.3× their width, respectively; length of inner hind spur 0.2× hind basitarsus; apex of hind tibia with distinct comb at inner side (Fig. 10).

***Metasoma*.** First tergite distinctly convex medially, as long as wide apically; first and second tergites with medio-longitudinal carina, weakly indicated on third tergite; first tergite densely longitudinally rugose; second and third tergites more or less obliquely rugulose (Fig. 8); medio-basal area of second tergite triangular and minute (Fig. 8); second suture deep and distinctly crenulate; remainder of metasoma superficially micro-sculptured or smooth; fourth and apical half of third tergite without sharp lateral crease; ovipositor sheath widened, with medium-sized slanted setae and apically subtruncate (Fig. 10).

***Colour*.** Dark brown; palpi, legs (but base of hind tibia dark brown), mandible (except dark brown teeth), malar space, clypeus and tegulae pale yellowish; orbita, propleuron, side of pronotum, mesosternum anteriorly, scutellum largely, first tergite medio-apically, second tergite medially (area widened posteriorly) and third tergite antero-medially yellowish brown; antenna, veins and pterostigma (but slightly paler basally than medially) mainly dark brown; third-sixth tergites posteriorly and laterally ivory (Figs 3, 8); wing membrane subhyaline, but surroundings of veins 1-M, 1-SR, 1-CU1 and r of fore wing more or less infuscate (Figs 5, 18).

##### Distribution

(from type material involved in this study): Austria, Belgium, Bulgaria, Czech Republic, England, Estonia, France, Finland, Germany, Hungary, Ireland, Italy, Netherlands, Norway, Serbia, Slovenia, Spain, Sweden, Switzerland.

##### Etymology.

The species is named “*pseudoseriatus*”, because of its similarity to *A.seriatus*.

##### Variation.

Pterostigma colour is rather variable, often with indistinct pale yellowish patch or entirely brown antero-basally, but sometimes with distinct yellowish basal patch; hind femur of ♀ usually 4.7–5.5 times longer than wide; ♀ with 46(1), 47(3), 48(8), 49(15), 50(13), 51(1) antennal segments and ♂ with 48(1), 50(2), 51(5), 52(14), 53(14), 54(11), 55(5), 56(4), 58(1) antennal segments; fourth antennal segment dark brown (Fig. 15), if brown then darker than scapus ventrally, rarely both are yellow; hind femur entirely yellowish brown or with faint brown small patch to large dark brownish part; metasoma with typical black pattern. Specimens with almost unmarked metasoma seem to occur very rarely or possibly not at all. Males have, on average, about three or four more antennal segments than females.

#### ﻿ Aleiodes seriatus

Taxon classificationAnimalia

(Herrich-Schäffer, 1838)

CAED36F6-80F6-53E3-A146-5D2739AFB5E0

Figs 21–25 , 26, 27 , 28–29 (see also figs 328–340 in van Achterberg and Shaw (2016))

Rogas seriatus Herrich-Schäffer, 1838: 156–12, fig. [type series lost].
Aleiodes seriatus ; Papp 1991: 107; Belokobylskij et al. 2003: 399.
Aleiodes vittiger Wesmael, 1838: 112; Shenefelt 1975: 1185; Papp 1991: 107; Belokobylskij et al. 2003: 399 (as synonym of A.seriatus) [examined].
Rogas kuslitzkyi Tobias, 1976: 88, 223–224; 1986: 83 (1995 transl.: 137).
Aleiodes kuslitzkyi ; Belokobylskij et al. 2003: 399 (as synonym of A.seriatus).

##### Type material.

The type series of *Aleiodesseriatus* (Herrich-Schäffer) is lost; as are the types of other Braconidae described by Herrich-Schäffer (Horn and Kahle 1935–1937; CvA could not find any specimen in the Zoological Museum in Berlin). The original description is rudimentary, and the figure shows only the colour pattern (which is highly variable) and there is a cryptic species in Europe. Considering the description (distinct yellowish base of the pterostigma), origin of the type series (assumed to be collected in the surroundings of Regensburg, Bavaria (his residence)) and its similarity with the lectotype of *A.vittiger*, this lectotype (♀, Royal Belgian Institute of Natural Sciences, Brussels, “*A.vittiger*, ♀, mihi, 13” (in Wesmael’s handwriting), “*A.vittiger* mihi, dét. C. Wesmael”, “Coll. Wesmael”, “Belgique, Bruxelles”, “Lectotypus ♀ *Aleiodesvittiger* Wesm., 1838, Papp, 1983”) is herewith designated as the neotype of *A.seriatus* (Herrich-Schäffer, 1838) to stabilize the taxonomy of the nominal species *A.seriatus* and *A.vittiger*.

##### Molecular data.

We have barcoded specimens from Albania (Gjurokaster), Bulgaria (Godech), England (Cambridgeshire), France (Ardèche, Corsica, Côte-d’Or, Dordogne, Var), Greece (Meteora), Italy (Veneto), Lithuania (Cepheliai), North Macedonia (Vardar), Serbia (Dukat, Suva Planina) and Spain (Mallorca: S’Albufera) (see Figs 1, 2).

##### Additional material.

Austria, Czech Republic, Netherlands (DR: Borger, Wijster, LI: St. Pietersberg, NB: Tilburg (Kaaistoep), Oisterwijk), Germany, Hungary, Montenegro, Poland, Russia, Sweden, Turkey.

##### Diagnosis.

Subbasal cell of fore wing setose apically (aa in Fig. 21; in ca 80% of ♀ specimens, 50% of ♂); pterostigma often distinctly pale yellowish antero-basally (Figs 22, 28, 29); hind femur of ♀ 5.3–6.0 times longer than wide (in ♂ up to 7.2 times); **if** (♀) hind femur partly dark brown laterally then also so ventrally; fourth antennal segment brown or yellowish brown ventrally, similar to scapus (Fig. 28); vein 1-M of fore wing of ♂ and surrounding area often less darkened than in *A.pseudoseriatus*.

**Figures 26, 27.**
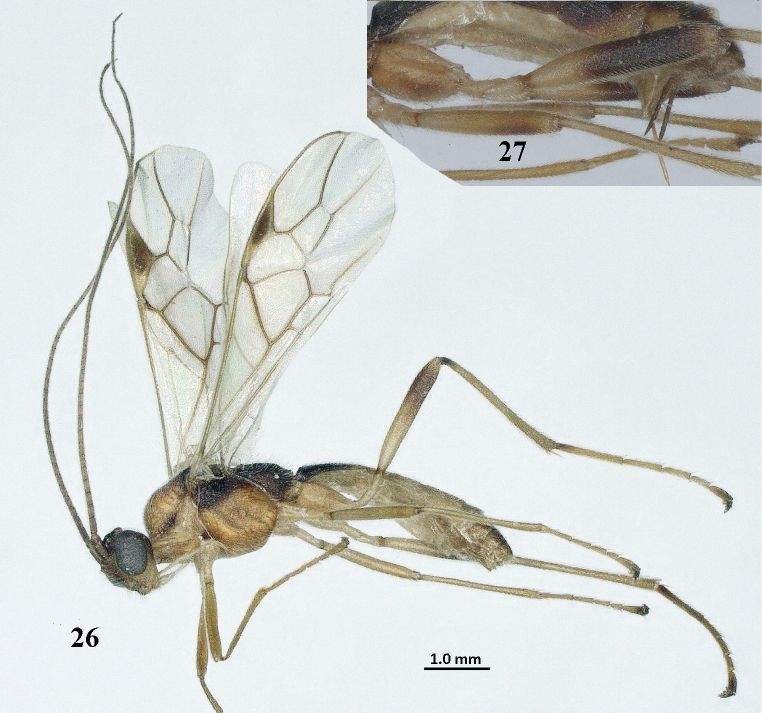
*Aleiodesseriatus* (Herrich-Schäffer), ♂ (**26**) and ♀ (**27**), England, Chippenham Fen. **26** habitus, lateral view **27** detail of hind femur and ovipositor sheath.

**Figures 28, 29.**
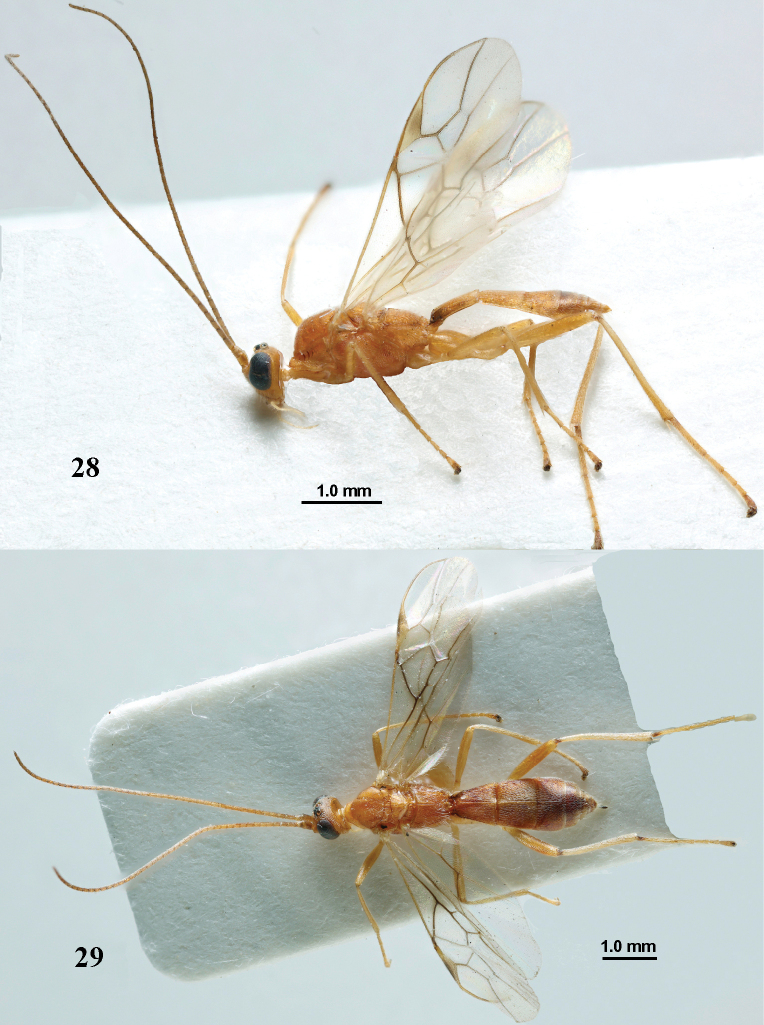
*Aleiodesseriatus* (Herrich-Schäffer) pale form, ♂ (**28**) and ♀ (**29**), France, Taradeau. **28** habitus lateral **29** habitus dorsal.

##### Variation.

Antenna of ♀ with 44(2), 45(10), 46(16), 47(20), 48(16), 49(5), 50(2), 51(1) antennal segments and of ♂ 46(2), 47(5), 48(8), 49(19), 50(16), 51(10), 52(4), 53(6), 54(2),55(4) segments. Males have, on average, about three or four more antennal segments than females.

##### Biology.

The only reared specimen seen is a male, accompanied by the host mummy, labelled as from *Lithosiagriseola* (= *Eilemagriseola* (Hübner), Lepidoptera: Erebidae, Arctiinae, Lithosiini) with the date 23/6.[19]33 from Hatert (Netherlands), in the E. Bauer collection (ZSM). The mummy is compatible, but it is unclear whether the date recorded is of collection or emergence, though probably the latter – but the rearing might nevertheless have been artificially advanced indoors. The host overwinters as a small larva, and presumably the parasitoid does so as an early instar larva inside the living host. It is notable that this increasingly widespread moth is found especially in wet woodland, fen carr, etc., and we have seen a long series of *A.seriatus* trapped in such places: Chippenham Fen, England (in NMS), and Černiš wetland, near České Budéjovice, Czech Republic (in IECB). We have also seen a female specimen (in E. Bauer collection, ZSM) reared in 1927 in the Netherlands labelled as coming from *Malacosomaneustria* (= *Malacosomaneustria* Linnaeus, Lepidoptera: Lasiocampidae) but there is no mummy present and we discount this as a credible record, not least on the grounds that this moth has a conspicuous and commonly reared caterpillar from which there are no further recorded rearings of *A.seriatus* (which, at least as an aggregate, is a distinctive entity likely to have been recorded). While capture dates mostly suggest a flight period of June to August into September, we have seen five specimens (including four males) collected in October – as well as a further eight males taken in September. These late males rather strongly suggest that there may be a (perhaps only partial) second generation, raising the possibility that a succession of *Eilema* species, with differing phenology, might constitute the host repertoire overall.

##### Distribution

(from material involved in this study): Albania, Austria, Bulgaria, Czech Republic, England, France (including Corsica), Germany, Greece, Hungary, Italy, Lithuania, Montenegro, Netherlands, North Macedonia, Poland, Russia, Serbia, Spain (Mallorca), Sweden and Turkey.

## ﻿Discussion

In common with most Lithosiini, the known hosts of the two *Aleiodes* species treated here are both becoming increasingly widespread and abundant in Europe, perhaps due to the recent change in atmospheric pollutants from a burden of sulphur dioxide, highly deleterious for algae, to increased levels of nitrogen oxides which encourage algal growth on aerial twigs. Probably the two *Aleiodes* species will prove to co-occur in an increasing number of localities.

In the tree, there are two sequences, MRS263 and MRS264, of female specimens that are morphologically indistinguishable from *A.pseudoseriatus* but cluster separately from it. While we acknowledge that there may be several reasons for this, they were collected at a site in France, Côte-d’Or, alongside specimens of both *A.seriatus* and *A.pseudoseriatus* (both barcoded) and, because we have found that other close *Aleiodes* species do sometimes hybridize in culture and produce female offspring (in prep.), we consider it possible that they are hybrids, albeit of unknown fitness.

*Rogaskuslitzkyi* Tobias, 1976 was synonymized with *A.seriatus* by Belokobylskij et al. (2003). At first, we believed that the sequences MRS239 and MRS241 of specimens from Primorsky Krai in the Russian Far East might belong to *A.kuslitzkyi*, which we have not been able to distinguish reliably from *A.pseudoseriatus* or *A.seriatus*. However, *A.kuslitzkyi* was described from the Caucasus region which is much closer to Europe than to the Far East of Russia. Considering the region and the colour of the pterostigma, it seems most likely that *A.kuslitzkyi* is indeed a synonym of *A.seriatus* as published by Belokobylskij et al. (2003) and that the Far East Russian (together with NW Chinese specimens we have seen elsewhere) belong to another new species. The number of antennal segments of both female types of *A.kuslitzkyi* (45, 47) also fits in with *A.seriatus* and excludes *A.pseudoseriatus*.

## Supplementary Material

XML Treatment for Aleiodes pseudoseriatus

XML Treatment for Aleiodes seriatus

## ﻿Additional information

### Author ORCIDs

Cornelis van Achterberg https://orcid.org/0000-0002-6495-4853

Mark R. Shaw https://orcid.org/0000-0002-6651-8801

Jose Fernandez-Triana https://orcid.org/0000-0003-0425-0309

### Data availability

All of the data that support the findings of this study are available in the main text.
